# Harnessing Nanotopography to Enhance Osseointegration of Clinical Orthopedic Titanium Implants—An *in Vitro* and *in Vivo* Analysis

**DOI:** 10.3389/fbioe.2018.00044

**Published:** 2018-04-11

**Authors:** Vitali Goriainov, Gry Hulsart-Billstrom, Terje Sjostrom, Douglas G. Dunlop, Bo Su, Richard O. C. Oreffo

**Affiliations:** ^1^Bone and Joint Research Group, Centre for Human Development, Stem Cells and Regeneration, Human Development and Health, Institute of Developmental Sciences, University of Southampton, Southampton, United Kingdom; ^2^Oral and Dental Sciences, University of Bristol, Bristol, United Kingdom

**Keywords:** surface topography, endosseous implants, orthopedic surgery, skeletal stem cells, osteogenesis

## Abstract

Despite technological advancements, further innovations in the field of orthopedics and bone regeneration are essential to meet the rising demands of an increasing aging population and associated issues of disease, injury and trauma. Nanotopography provides new opportunities for novel implant surface modifications and promises to deliver further improvements in implant performance. However, the technical complexities of nanotopography fabrication and surface analysis have precluded identification of the optimal surface features to trigger osteogenesis. We herein detail the osteoinductive potential of discrete nanodot and nanowire nanotopographies. We have examined the ability of modified titanium and titanium alloy (Ti64) surfaces to induce bone-specific gene activation and extracellular matrix protein expression in human skeletal stem cells (SSCs) *in vitro*, and *de novo* osteogenic response within a murine calvarial model *in vivo*. This study provides evidence of enhanced osteogenic response to nanowires 300 surface modifications, with important implications for clinical orthopedic application.

## Introduction

Medical and technological advances have led to a welcome increase in life expectancy, and yet the loss or dysfunction of skeletal tissue that can accompany trauma, injury, disease or advancing years can result in significant morbidity as well as a variety of socio-economic issues. Indeed the 2000-2010 decade was endorsed by the WHO and the UN as the Bone and Joint Decade, acknowledging the major burden of musculoskeletal (MSK) disease. Osteoarthritis (OA) is one of the most prevalent MSK disorders (Woolf, 2012), affecting 9.6% of men and 18% of women aged over 60 years (Murray and Lopez, 1996). The impacts of OA on an individual patients' quality of life and health care system in general are anticipated to intensify with the aging population and higher prevalence in older groups (Woolf and Pfleger, 2003). Despite the prevailing success of hip arthroplasty in treatment of hip osteoarthritis (Learmonth et al., 2007), 11% of patients remain dissatisfied at 1 year following hip replacements (RCSEng, 2000; Garellick et al., 2010). As the largest arthroplasty database in the world, the UK National Joint Registry shows overall hip replacement revision rates of 6.46% at 12 years, and 3.93 and 8.37% revision rates of cemented and uncemented implants, respectively (NJR, 2016). Thus, the intuitive desire to develop for patient application a biological implant/bone interface able to dynamically respond and self-repair under the changing physiological demands remains a key unmet orthopedic need. The establishment of an appropriate biological implant-bone interface depends on the process of osseointegration, which, in turn, is reliant upon establishment of primary and subsequently secondary stability. Secondary stability is achieved by bone in- or on-growth on the implant surface as a result of de novo osteogenesis. Implant surface properties have been shown to regulate the process of contact osteogenesis (Goriainov et al., 2014), responsible for a series of events ranging from implant surface colonization by skeletal stem cells (SSCs) to appositional bone formation (Davies, 2003).

The development of surfaces able to augment implant integration with host tissue is therefore more than research curiosity. This process should involve modifications of the existing surface finishes, with particular focus on addressing specific nanotexture-enabled properties impacting cellular level interactions. Meticulous monitoring of clinical outcomes following the surface modifications is warranted. The ability of an uncemented implant to attain full implant/bone contact will enable reduction of transferred mechanical load density through maximizing the area of contact with the elimination of interface deficiencies. This would preclude wear particle entry into the implant/bone interface and subsequent inflammatory-mediated osteolytic processes (Abu-Amer et al., 2007). In turn, these factors promise to prevent at least 24.3% of hip revision and 29.8% of knee revision procedures that are currently undertaken for aseptic loosening, which in real numbers amounts to over 4,000 cases per year in the UK alone (NJR, 2013; Khan et al., 2016).

The importance of the concept that surface structure could be pivotal in modulating cell orientation and contact guidance was recognized in the early twentieth Century (Harrison, 1911); while the seminal work of Curtis and Varde demonstrated the importance of topography for the cell and the concept of contact guidance and guidance-cell environment (Curtis and Varde, 1964). Indeed, the stimulation of an enhanced SSC-mediated osteogenic response by implant surface topography has been extensively investigated (Goriainov et al., 2014). A number of investigations using materials developed at the micro-scale and nanoscale topographies have been shown to trigger an enhanced osteogenic response (Zhao et al., 2005; Dalby et al., 2008; Sjöström et al., 2009; Olivares-Navarrete et al., 2012; Zhang et al., 2012; Qian et al., 2017; Xiao et al., 2017) and, furthermore, there is a wealth of literature on the ability of nanotopographical substrates to modulate SSC differentiation, maintain SSC phenotype or differentiate human embryonic stem cells along the mesoderm lineage (Dalby et al., 2006, 2014; Hollander et al., 2006; Gittens et al., 2011, 2014; McMurray et al., 2011; Kingham and Oreffo, 2013; Kingham et al., 2013; Goriainov et al., 2014; Wang X. et al., 2016). Ti surface-modifying technologies result in a range of surface parameters, which in turn trigger heterogeneous SSC responses (Goriainov et al., 2014). However, the optimal topographical features have, to date, not been identified.

Aseptic loosening and infection remain the two leading causes of orthopedic implant failures (NJR, 2016). We have previously demonstrated the nanowire surface textures fabricated by the thermal oxidation method exhibiting bactericidal properties, a highly desirable property for an implant surface (Sjostrom et al., 2016). In the current study, aiming to address implant aseptic loosening through formation of enhanced implant/bone interface, we explored the osteoinductive potential of the equivalent nanowire surface topographies by examining their *in vitro* and *in vivo* biological interactions with human SSCs. We have analyzed the ability of generated topographies to induce SSC osteogenic lineage differentiation *in vitro*, as measured by morphological alterations, osteogenic gene expression and bone-specific extracellular protein synthesis. This SSC-mediated biological response was compared to other complex nanotopographical surfaces fabricated using anodizing and thermal oxidation techniques. Subsequently, the *in vivo* outcomes on nanowires were analyzed by measuring appositional de novo osteogenic response in a calvarial rat model.

## Materials and methods

### Materials, substrate preparation, and characterization

**Figure 1:**
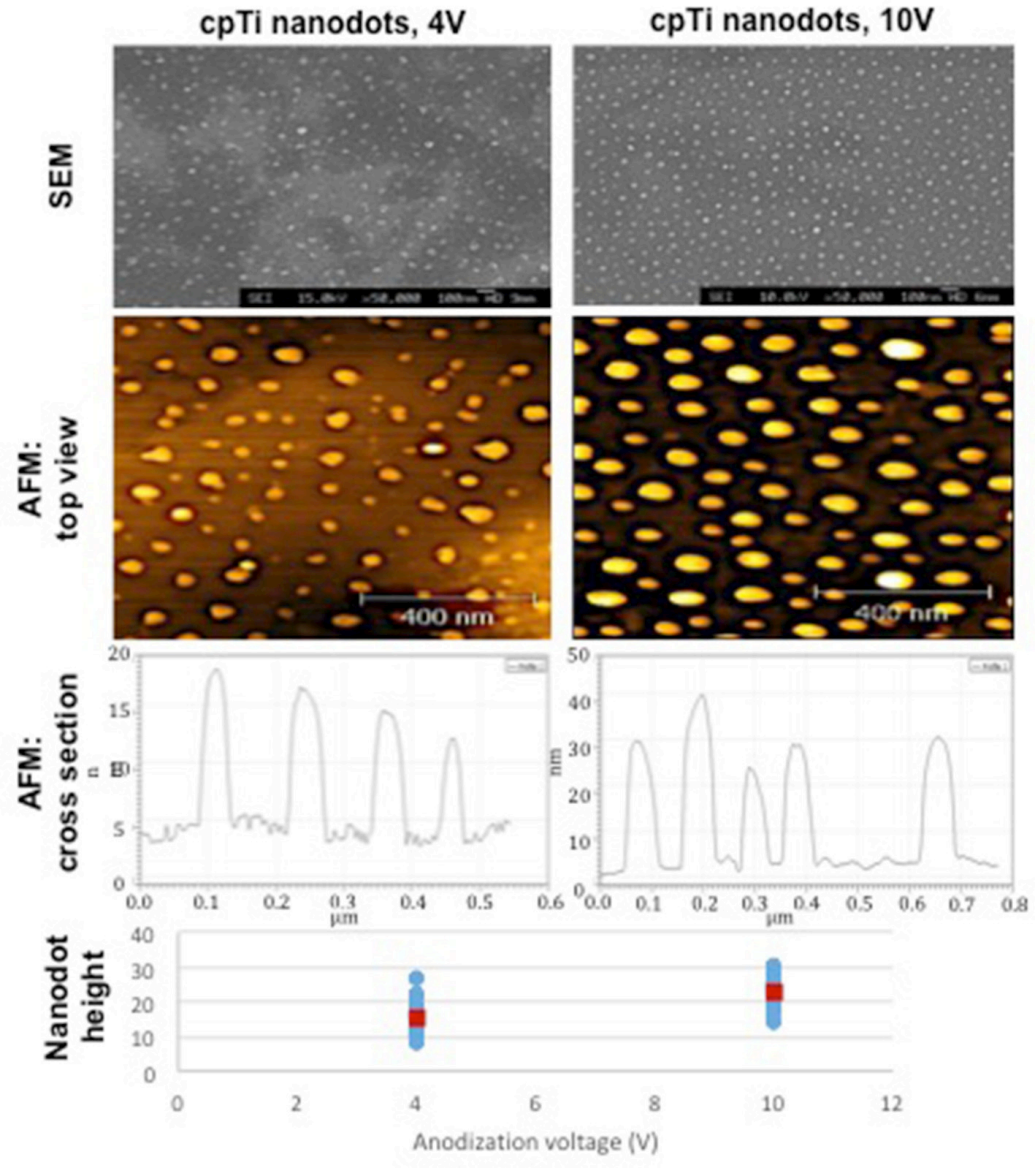
The diameter, height and surface distribution pattern of nanodots fabricated using an anodization protocol. The heights of individual nanodots on the bottom chart were taken from tapping mode AFM data [atomic force microscope (Veeco Multimode with Quadrex Nanoscope 3D)]. Blue markers show individual measurements, red markers show the average nanodot height [nanodots 4 V−15 nm (8–27), nanodots 10 V−22 nm (13–31)].

Fourteen mm diameter discs and 10 mm squares were cut from a chemically pure titanium (cpTi) sheet and a Ti6Al4V (Ti64) sheet (Titanium Metals Ltd), respectively. The samples were polished to a mirror shine (TegraPol-15, Struers), sonicated in acetone for 10 min and air-dried. For TiO2 nanodot patterning, an anodization method using a block copolymer template was applied (Sjöström et al., 2012). Mirror polished cpTi samples were coated with a thin film of polystyrene-b-poly 4-vinylpyridine (PS-b-P4VP) block copolymer (Polymer Source, molecular weight 480k-b-150k) using a spin coater. The PS-b-P4VP films were solvent annealed in a tetrahydrofuran (THF) atmosphere for 3 h. The samples were anodized in 0.01 M oxalic acid at room temperature with a voltage of 4 or 10 V, respectively. After anodization, the PS-b-P4VP thin film templates were removed by O_2_ plasma treatment at 150 W for 40 min. The resultant TiO_2_ nanodots are depicted in Figure 1.

**Figure 2:**
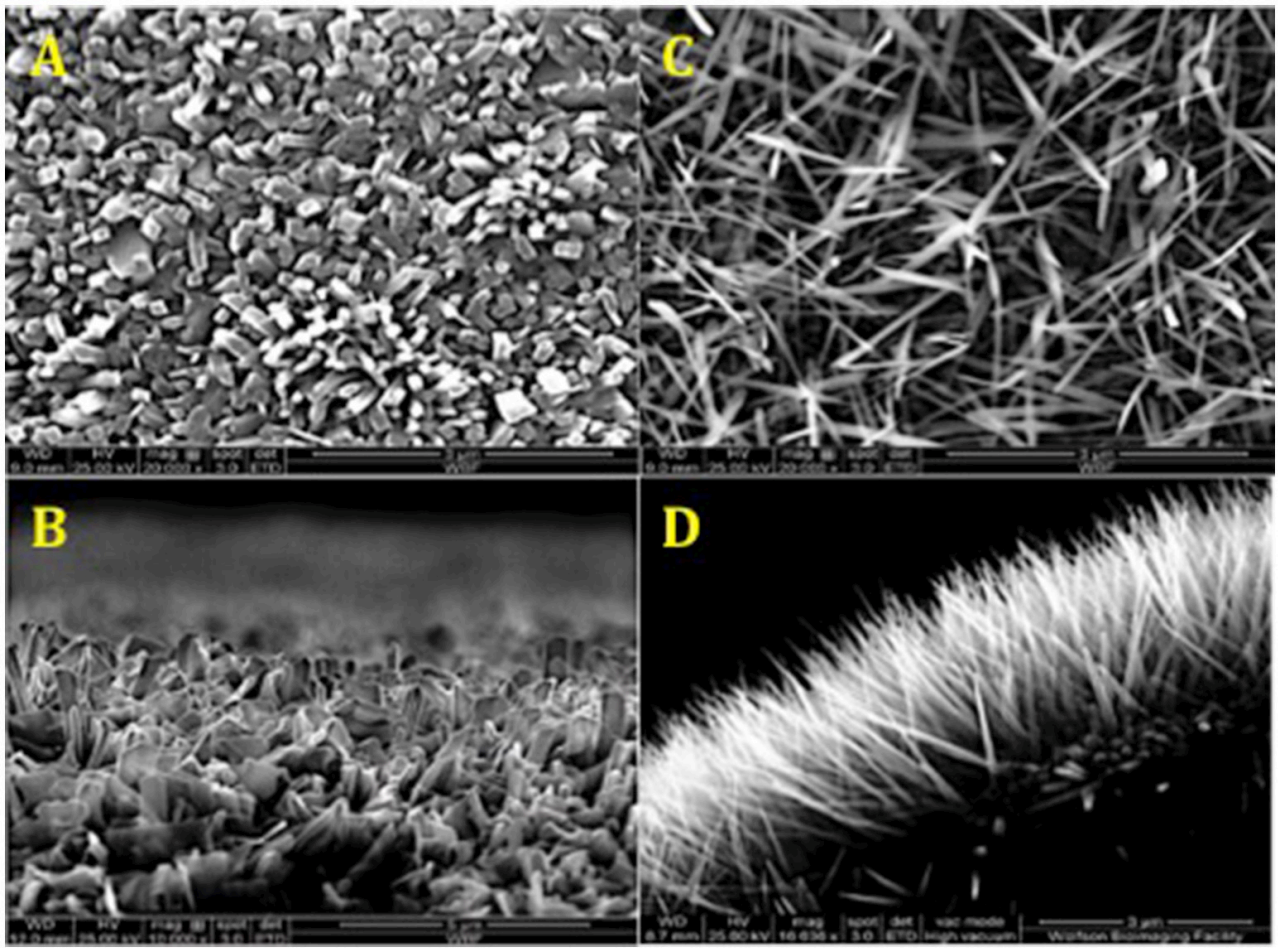
SEM of surface topographies generated by thermal oxidation method. **(A,C)** Top view TiO_2_ nanowires 50 and 300 sccm, respectively. **(B,D)** Side view TiO_2_ nanowires 50 and 300 sccm, respectively. The average length of TiO_2_ nanowire 300 sccm spikes on the surface was 3 μm, and the average diameter was 20 nm.

For TiO2 nanowire patterning, a controlled thermal oxidation method was used (Sjostrom et al., 2016). Ti6Al4V alloy (Ti64) samples were heat-treated in a horizontal alumina tube furnace (1,500 mm long, 95 mm inner diameter). After purging the tube with Ar, the temperature was increased to 850°C at 15°C/min. After reaching 850°C the Ar flow was diverted through a bubbler bottle containing acetone at 25°C, with the Ar flow rate adjusted to 50 or 300 sccm for 30 min. Tube was then allowed to cool to room temperature under the flow of Ar at 500 sccm. Subsequently, the samples were annealed at 600°C for 30 min in air to remove carbon from the surfaces, resulting in formation of TiO2 nanowires seen in Figure 2. A JEOL JSM 5600LV field emission scanning electron microscope (SEM) was used to image the oxidized Ti64 surfaces as previously described (Sjostrom et al., 2016).

Two substrate groups were formed based on the bulk material (Table 1). Each group was sub-divided into a further three groups based on surface characteristics. In subsequent experiments each group had an internal control (planar surface in both *in vitro* and *in vivo*), as well as an external control (TCP in *in vitro* testing only).

**Table 1:** The test groups generated as a result of employing different bulk materials and surface treatments.

**Bulk material**	**Surface finish**
cpTi	Planar
	Nanodots 4 V
	Nanodots 10 V
TiAl_6_V_4_	Planar
	Nanowires 50 sccm (Nanowires 50)
	Nanowires 300 sccm (Nanowires 300)

### Human skeletal stem cell (SSC) cell culture

Adult human osteoprogenitor and SSC populations were enriched from femoral head samples and bone marrow obtained from hematologically healthy patients undergoing hip replacement surgery with local ethics committee approval (LREC194/99/1), as previously described (Yang et al., 2003). Enrichment of SSC fraction from bone marrow cell population was achieved using magnetic sorting system and Stro-1 antibody, as previously described (Howard et al., 2002; Mirmalek-Sani et al., 2006). Primary cultures were established from 3 physiologically healthy donors: (mean age 72.7 years; 2 females 78 and 71 years of age and 1 male 69 years of age). All 3 donors demonstrated consistent viability and cell growth.

SSCs were cultured in a basal medium (α-MEM/10% FCS/1% P/S) at 37°C in 5% CO_2_, with medium changes performed twice a week. Individual experiments were performed using exclusively passage 1 cells from discrete patient donors unadjusted for demographics (3 donors for 3 repeats of *in vitro* experiments). SSCs were seeded at 220 cells/cm^2^ density either directly onto substrates (test groups) or TCP (control groups) for subsequent 21 day *in vitro* cultures.

Preparation of substrates was undertaken in PBS/1% antibiotic-antimycotic solution (Life Technologies, Invitrogen) for a minimum of 24 h, before transfer into culture plates and a PBS wash prior to cell seeding or *in vivo* implantation.

### Live/dead cell assay

Cell viability and morphology were evaluated using CellTracker™ Green (CTG) CMFDA and ethidium homodimer-1 (Life Technologies, Invitrogen). Fifty microgram of CTG and 5 μg of ethidium homodimer were dissolved in 10 μl of DMSO prior to addition to the culture medium.

Following *in vitro* culture, substrates with adherent cells were washed in PBS and fixed overnight in 3% glutaraldehyde, 4% paraformaldehyde and 0.1 M PIPES (Sigma-Aldrich). Subsequently, the samples were dehydrated through graded ethanol (30, 50, 70, 90, and 100%) and dried. The samples were sputter coated with platinum to achieve a 7 nm layer thickness prior to imaging.

### *In vitro* immunocytochemistry

After 3 weeks *in vitro* culture, cells adherent to substrate surfaces were rinsed in PBS and fixed in 4% PFA, blocked with bovine serum (Sigma-Aldrich), treated overnight in anti-OPN or anti-Collagen type I αI primary antibody raised in rabbit (GeneTex), followed by goat anti-rabbit IgG (H+L) secondary antibody, Alexa Fluor® 488 conjugate (Sigma-Aldrich). Nuclear counterstaining was performed using DAPI (4′,6-Diamidino-2-Phenylindole, Dihydrochloride) (Life Technologies, Invitrogen). The substrates were mounted on slides and imaged using Zeiss Axiovert 200 inverted microscope.

Following fixation in 4% PFA and bovine serum block, cytoskeletal immunostaining of actin microfilaments was performed by incubating cells at room temperature with Alexa Fluor 647 Phalloidin conjugate (ThermoFisher Scientific). The samples were counterstained using DAPI and subsequently mounted.

### Molecular analysis of osteogenic gene expression

Trypsin-EDTA buffer (Sigma-Aldrich) was used to release cells from culture surfaces (8 material replicates) prior to lysis. Total mRNA extraction was accomplished using the Qiagen RNeasy kit in accordance with manufacturer's instructions. mRNA samples were treated with DNAse and reverse-transcribed using SuperScript first-strand synthesis system (Veriti Thermal Cycler, Applied Biosystems). Real-time qPCR using SYBR® Select Master Mix (Life Technologies) was carried out on 7500 Real-Time PCR system (Applied Biosystems) for amplification of β-actin, ALP, Collagen type I, OPN, OCN and Collagen type II genes. β-actin was employed as the house-keeping gene and Collagen type II as a negative control. Primer sequences (Table 2) were validated by dissociation curve/melt curve analysis and efficiencies of amplification for the β-actin primers and primers for the bone marker genes of interest were approximately equal. The quantification of PCR amplification data was achieved using comparative cycle threshold method and relative transcript levels were expressed as mean ± S.D. Data were analyzed and plotted using GraphPad Prism 6 for Mac OS X software.

**Table 2:** Primer sequences used for RT-PCR.

**Gene**	**Primer pairs**	**Amplicon**	
β-Actin	F: 5′ GGC ATC CTC ACC CTG AAG TA 3′	82	NM_001101
	R: 5′ AGG TGT GGT GCC AGA TTT TC 3′		
ALP	F: 5′ GGA ACT CCT GAC CCT TGA CC 3′	86	NM_000478
	R: 5′ TCC TGT TCA GCT CGT ACT GC 3′		
Collagen type IαI	F: 5′ GAG TGC TGT CCC GTC TGC 3′	52	NM_000088
	R: 5′ TTT CTT GGT CGG TGG GTG 3′		
OPN	F: 5′ GTT TCG CAG ACC TGA CAT CC 3′	80	NM_001251830
	R: 5′ CAT TCA ACT CCT CGC TTT CC 3′		
OCN	F: 5′ GGC AGC GAG GTA GTG AAG AG 3′	102	NM_001199662
	R: 5′ CTC ACA CAC CTC CCT CCT 3′		
Collagen type IIαI	F: 5′ CCT GGT CCC CCT GGT CTT GG 3′		
	R: 5′ CAT CAA ATC CTC CAG CCA TC 3′		

### *In vivo* evaluation of implant substrates

**Figure 3:**
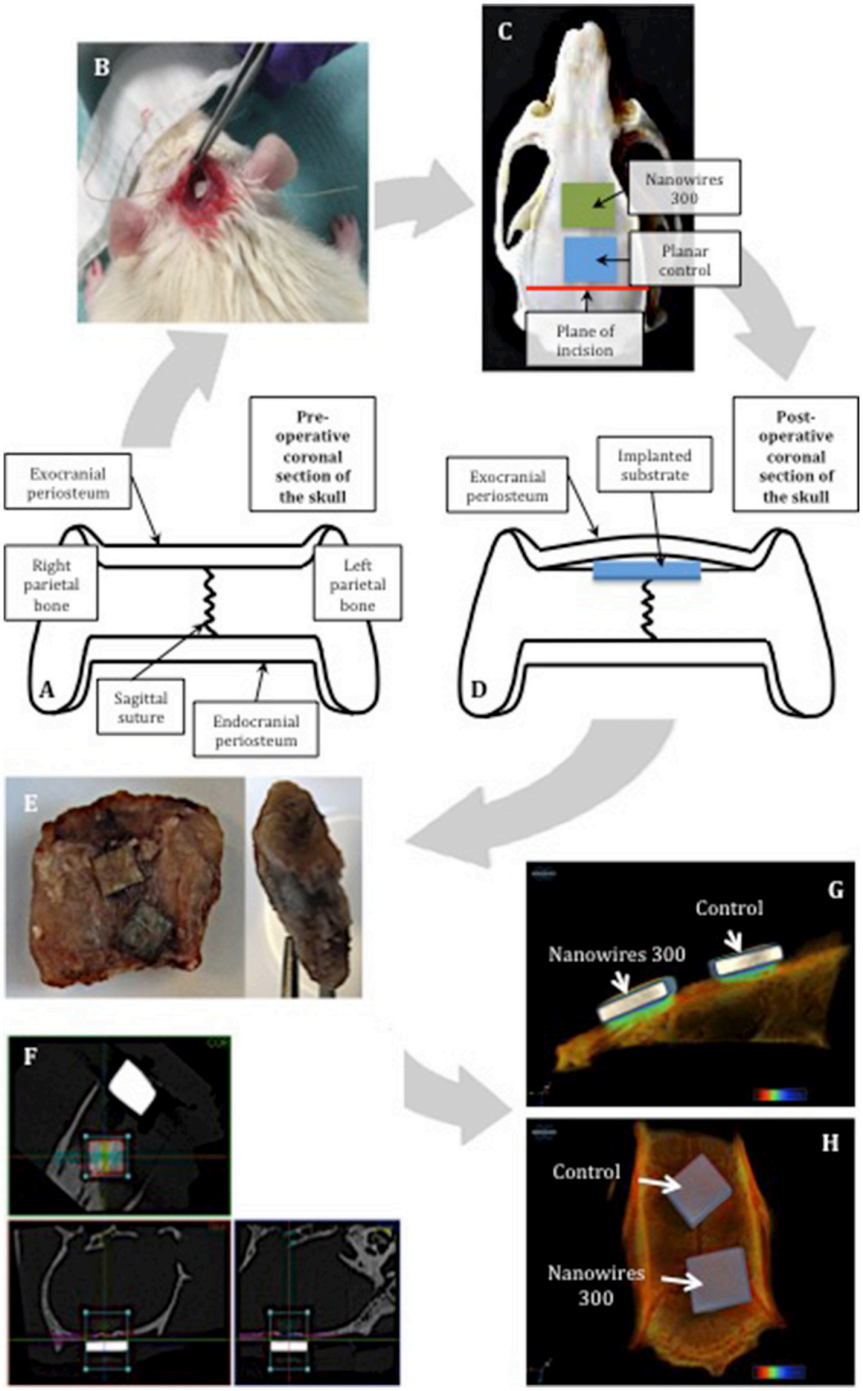
Schematic representing the use of the calvarial model for *in vivo* substrate culture. The natural flat plane over the superior parts of parietal bones was laterally confined by bilateral supraorbital ridges **(A)**. The substrates were implanted using minimally invasive technique **(B)**. Restraint was created by the overlying exocranial periosteum, combined with friction with underlying feathered bone, enabling the implanted substrates to achieve primary stability **(C,D)**. The samples were removed with the underlying segment of calvarium and enveloped with overlying periosteum **(E)**. Manual alignment of nanowires 300 **(F)** samples along coronal, trans-axial and sagittal axes for CT scanning. 3D reconstructions of the samples following 8 weeks of *in vivo* culture: view from sagittal view **(G)** and the top **(H)**.

Two sample surfaces were selected for further *in vivo* testing: Planar as a control and nanowires 300 as a result of superior osteogenic potential demonstrated *in vitro*. Following appropriate preparation, one sample from each test group was implanted into male ex-breeder Sprague Dawley rats in accordance with the calvarial model protocol outlined in Figures 3A–D. The periosteal flap was elevated off the parietal bones of skull to create a pocket, the surface of the skull was then feathered to create a flat cancellous bed for substrate implantation, and the periosteal flap was closed above to secure the samples. While under anesthesia, the rats were transferred into a SkyScan 1176 (SkyScan, Bruker, Kontich, Belgium) for immediate post-operative scanning, before recovery. The scans were repeated at 2-weekly intervals post-operatively. At 8 weeks, the rats were euthanized, and the substrates and the underlying segments of parietal bones were retrieved for histological examination (Figure 3E).

### CT-based radiological evaluation of bone/implant interface

Individual specimen image data was acquired using the Skyscan 1176 (SkyScan, Bruker, Kontich, Belgium) immediately post-surgery (week 0) and at weeks 2, 4, 6, and 8. The following settings were used: source voltage−90 kV; current−278 μA; filter—Cu 0.1 mm; exposure time−165 ms; frame averaging−1; rotation step−0.7°; rotation−180°. Reconstruction of cross sections was undertaken using software package NRecon (SkyScan, Bruker, Kontich, Belgium) with automatic correction for misalignment, ring artifacts and beam hardening (40%). The alignment was further adjusted manually along three axes as shown in Figure 3F.

The region of interest (ROI), selected to include a region of the implant being in optimal contact with adjacent bone, was set to 2.5 × 1.25 mm in 75 consecutive cross sections, and included the whole thickness of an individual implant and underlying calvarial bone tissue. Thus, the bone volume analyzed included both the original calvarial tissue and any new bone formed adjacent to the sample surface. CT analysis was undertaken using CTAn (SkyScan, Bruker, Kontich, Belgium). Global thresholding was used to segment bone from the background. The resultant data was compiled using GraphPad Prism 6 for Mac OS X software to generate mean values of the temporal bone volume trend for individual sample group, which was later used for quantitative statistical comparison. Three-dimensional reconstructions of the samples (Figures 3G,H) were obtained using CTvox (SkyScan, Bruker, Kontich, Belgium).

### *In vivo* histological analysis

Following micro-CT analysis, the calvarial bones were retrieved and the segments immediately underlying the substrate surface topographies carefully dissected and fixed in 4% PFA, ensuring that correct orientation was preserved. The samples were decalcified in 5% EDTA, 0.1M Tris pH 7.3 for 21 days, dehydrated through a series of graded alcohols, embedded in low-melting point paraffin using automated Shandon Citadel 2000 and cut to obtain 7 μm thick sections. The sections were rehydrated and stained with 0.5% Alcian blue 8GX (for proteoglycan-rich cartilaginous matrix) and 1% Sirius red F3B (for collagenous bone matrix). Alternatively, the sections were permeabilized with 3% H_2_O_2_ (Sigma-Aldrich), and subsequent immunostaining with anti-OCN primary antibody raised in rabbit (GeneTex) followed the steps described in *in vitro* immunocytochemistry. All sections were subsequently dehydrated and cleared before mounting in DPX.

### Imaging

Image capture was undertaken with a Zeiss Axiovert 200 inverted microscope using an Axiocam MR camera for fluorescent imaging and Axiovert HR camera for white light imaging operated by Zeiss Axiovision software version 4.7. Alcian blue Sirius Red (A&S) histology sample imaging was performed with an Olympus BX-51/22 dotSlide digital virtual microscope using OlyVIA 2.1 software (Olympus Soft Imaging Solutions, GmBH). Scanning electron microscopy was performed on FEI Quanta 250 FEG SEM microscope using xT Microscope software.

### Statistical analysis

The statistical analysis was carried out using Microsoft Excel for Mac 2011 Version 14.6.4 (Microsoft Corporation) and Prism 6 for Mac OS X (GraphPad Software) with data presented as mean±standard deviations. *In vitro* experiments were repeated three times to ensure validity and reproducibility of the results. Eight independent culture samples were pooled for RT-PCR analysis to minimize the effect of the variation introduced by individual samples. The results were expressed as mean ± standard deviation (SD). Two-way ANOVA test was used in comparisons for multiple factors between seven independent test groups. *T*-test was used in comparison for a single factor between two independent groups. The significance level was set at *p* < 0.05.

## Results

### Evaluation of skeletal stem cell growth on nanosurfaces

**Figure 4:**
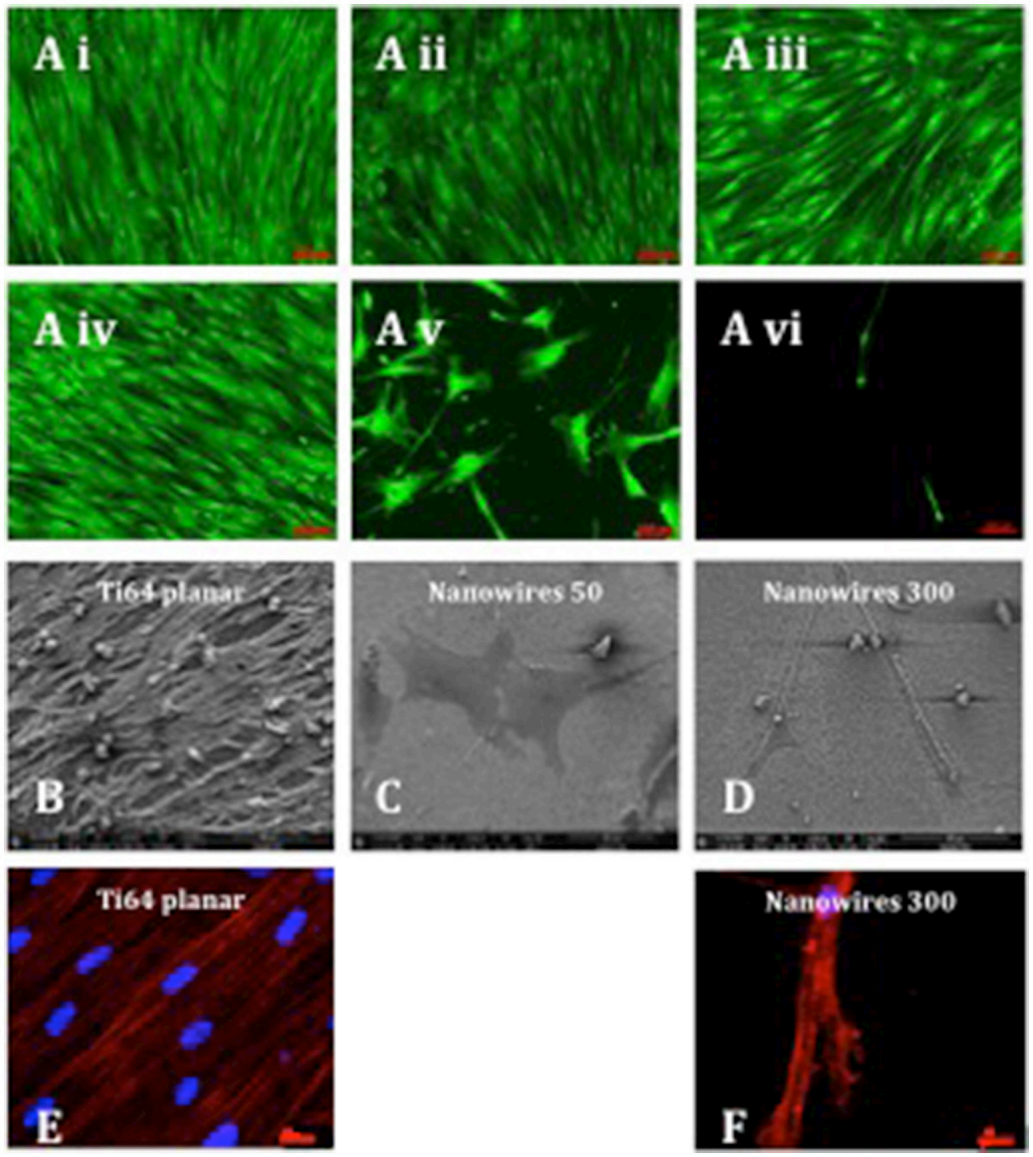
**(A)** Stro-1 SSC viability and morphology on different test surfaces **(Ai)** Ti planar, **(Aii)** Nanodots 4V, **(Aiii)** Nanodots 10V, **(Aiv)** Ti64 planar, **(Av)** Nanowires 50, **(Avi)** Nanowires 300) following 21 days of *in vitro* culture (scale bar for nanowire 300 represents 200 μm and other surfaces−100 μm). SSCs were seeded at 220 cells/cm^2^ density, and this density was maintained throughout the 21 days of *in vitro* culture, indicating minimal cell expansion as a consequence of early nanowire topography-mediated signaling and preferential SSC differentiation. **(B–D)**. SEM images of SSCs cultured on test surfaces *in vitro* for 21 days (scale bar 100 μm). Immunostaining of actin microfilaments following 21 days of *in vitro* culture on Ti64 planar surfaces (**E**—scale bar 20 μm) and nanowires 300 (**F**—scale bar 20 μm).

STRO-1 Enriched human SSCs were observed to proliferate, remain viable and to reach confluence after 21 days of *in vitro* culture on a range of substrates (Figure 4A). SSCs displayed an elongated and fibroblast-like morphology on planar surfaces irrespective of bulk material. Initial induction of a morphological osteoblastic-like (cuboidal) appearance was observed on nanodots 10 V. In contrast, SSCs maintained on nanowires 50, displayed an enhanced stellate osteoblast-like morphology, while on nanowires 300 the skeletal populations displayed a distinct teardrop-shaped morphology and marked extensions between several focal adhesion points. On nanowires 300, and to a lesser extent on nanowires 50, the density of viable cells was observed to be reduced although there was no evidence of significant cell apoptosis observed. On nanowires 300, SSCs displayed an increasingly distinct elongated morphology between days 7 and 21.

Scanning electron microscopy analysis of SSCs on Ti64 planar surfaces revealed the formation of a cell monolayer with extensive intercellular connections (Figure 4B). On nanowires 50, SSCs presented as a flat, spread, stellar appearance with cytoplasmic projections approaching 400 μm in length and fewer filopodia with pronounced actin stress fibers (Figure 4C). In contrast, the SSCs on nanowires 300 displayed a distinct elongated morphology with cytoplasmic projections approaching 400 μm in length and a more globular cell presentation with multiple fine adhesion filopodia that appeared to penetrate the nanowire lattice (Figure 4D). Given the low density of cells on nanowire 300 constructs, few intercellular connections observed in these cultures.

An interconnected and aligned cell monolayer was observed on Ti64 planar surfaces evidenced by Phalloidin immunostaining of filamentous actin. On nanowires 300, actin microfilaments were noted to be aligned and to form a cytoskeletal framework interlinking individual focal attachment complexes of the osteoprogenitor cells (Figures 4E,F).

### Osteogenic marker expression of SSCs following culture on nanosurfaces

**Figure 5:**
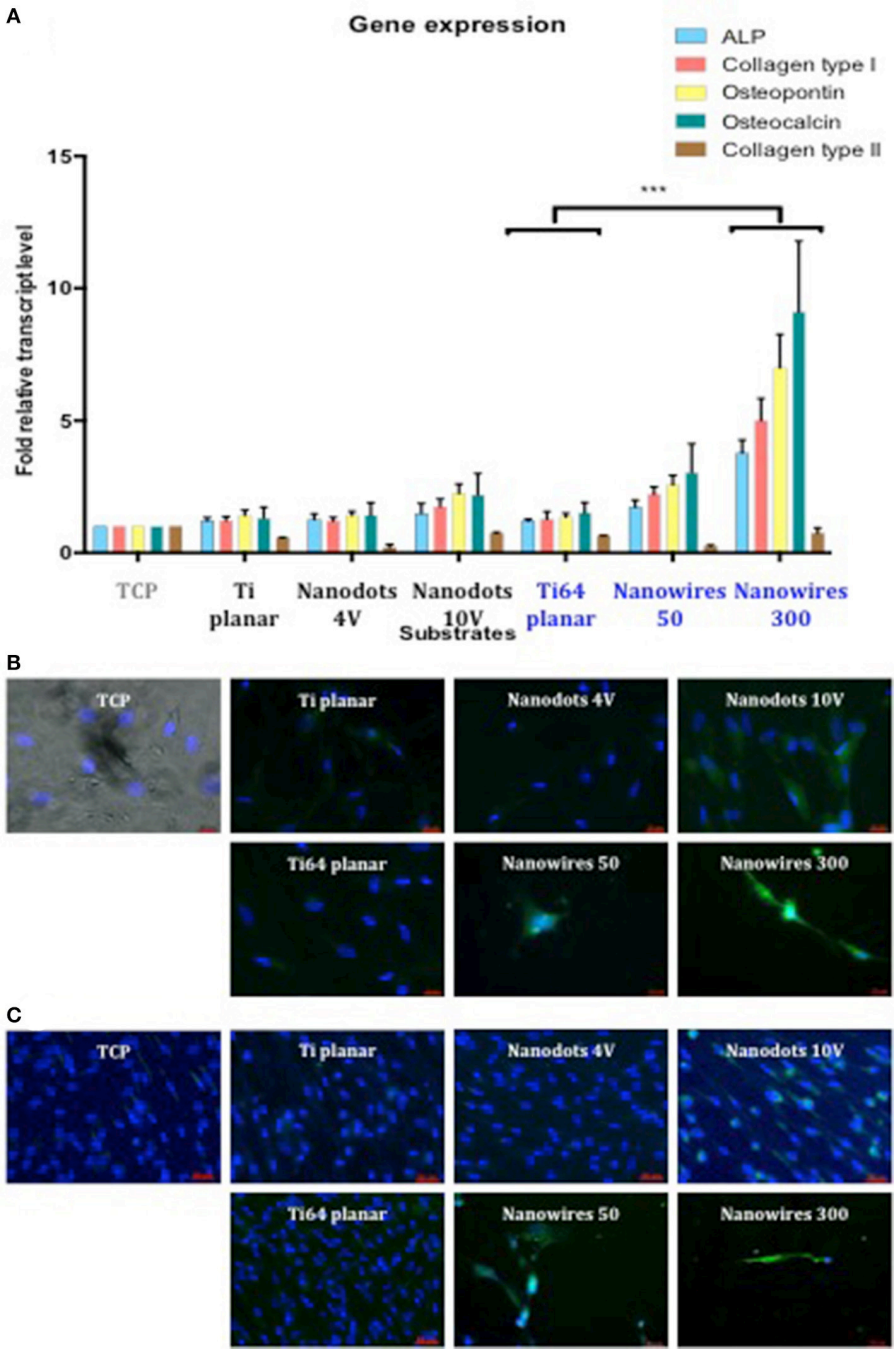
**(A)**. Real-time qPCR analysis of osteogenic genes (ALP, Collagen type I, OPN, and OCN) with the chondrogenic gene Collagen Type II as a negative control in STRO 1 SSCs cultured *in vitro* on test surfaces and tissue culture plastic for 21 days. TCP surface served as a reference point. Results expressed as mean ± SD, triplicate samples, individual experiment repeated three times, 2-way ANOVA test, ^***^p < 0.001. **(B)**. Fluorescent imaging of OPN immunostaining in SSCs after 21 days of *in vitro* cultures (blue—cell nuclei, green—OPN protein). Scale bar 20 μm. **(C)**. Fluorescent imaging of Collagen type I immunostaining in SSCs after 21 days of *in vitro* cultures (blue—cell nuclei, green—Collagen type I protein). Scale bar 50 μm.

SSCs cultured, *in vitro*, on Ti and Ti64 control surfaces demonstrated comparable levels of osteogenic gene induction for the following markers ALP, Collagen type I, OPN and OCN. Gene expression was enhanced on Ti and Ti64 substrates in comparison to TCP but this did not reach statistical significance (Figure 5A). SSCs cultured on nanowires 300 displayed significantly enhanced expression of osteogenic marker genes compared to control substrates. In contrast, Collagen type II gene expression, a marker of cartilaginous extracellular matrix, was significantly reduced on all test surfaces in comparison to SSCs maintained on TCP.

SSCs cultured on nanowires, in particular on nanowires 300, displayed significantly enhanced expression of OPN and Collagen type I immunofluorescence in comparison to expression on TCP and planar surface controls as shown in Figures 5B,C, respectively. Induction of OPN and Collagen type I expression was also observed on nanodots 10 V compared to planar surfaces, although the levels of expression were less marked in comparison to nanowires.

### Computer tomography (CT)-based analysis of *in vivo* nanosurfaces

**Figure 6:**
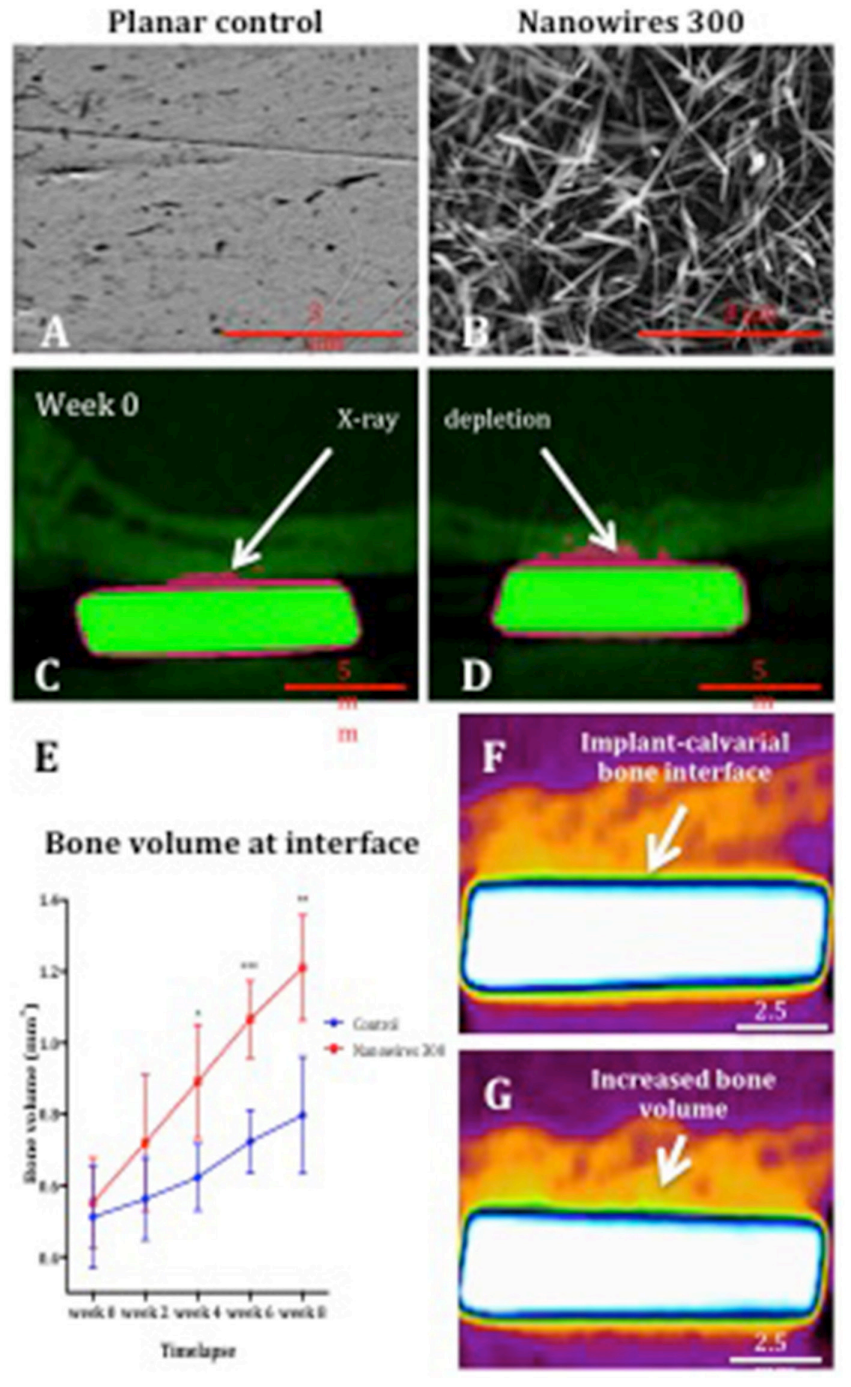
The SEM images of planar control **(A)** and nanowires 300 **(B)** test group surfaces, and coronal cross sections of control **(C)** and nanowires 300 **(D)** at week 0 post-operative CT. Note the samples (green) and the metal artifact (pink) produced as a result of beam hardening. **(E)** μCT analysis of bone volume at the interface with planar control and nanowires 300 sample surfaces measured at 2-weekly intervals over 8 weeks of *in vivo* culture. Results expressed as mean ± SD, *n* = 5, *t*-test, ^*^p < 0.05, ^**^p < 0.01, ^***^p < 0.001. Reconstructed images of the interface of a nanowires 300 sample and overlying calvarial bone from circa the same area of the interface at week 0 **(F)** and following 8 weeks **(G)** of *in vivo* culture. Note the increased bone volume (bright orange color) at the interface at 8 weeks.

Following planar control and nanowire 300 (Figures 6A,B) sample implantation in a rat calvarial model, the initial post-operative CT at week 0 indicated greater X-ray depletion by nanowires 300 samples, resulting in a larger metal shadow artifact (Figures 6C,D).

A significant increase in bone volume at the calvarial bone interface with nanowires 300 sample surface after 8 weeks of *in vivo* culture compared to control samples was observed (Figure 6E). A time-dependent increase in bone volume was observed in both experimental groups with the rate of bone volume increase significantly enhanced at the interface with nanowires 300 surfaces. Significant differences in the rate of bone formation were observed from 4 weeks of *in vivo* implantation. The enhanced effect of nanowires 300 surface topography on the bone volume at the adjacent interface was further visually confirmed by comparison of images at weeks 0 and 8 time points (Figures 6F,G).

### *In vivo* histology

**Figure 7:**
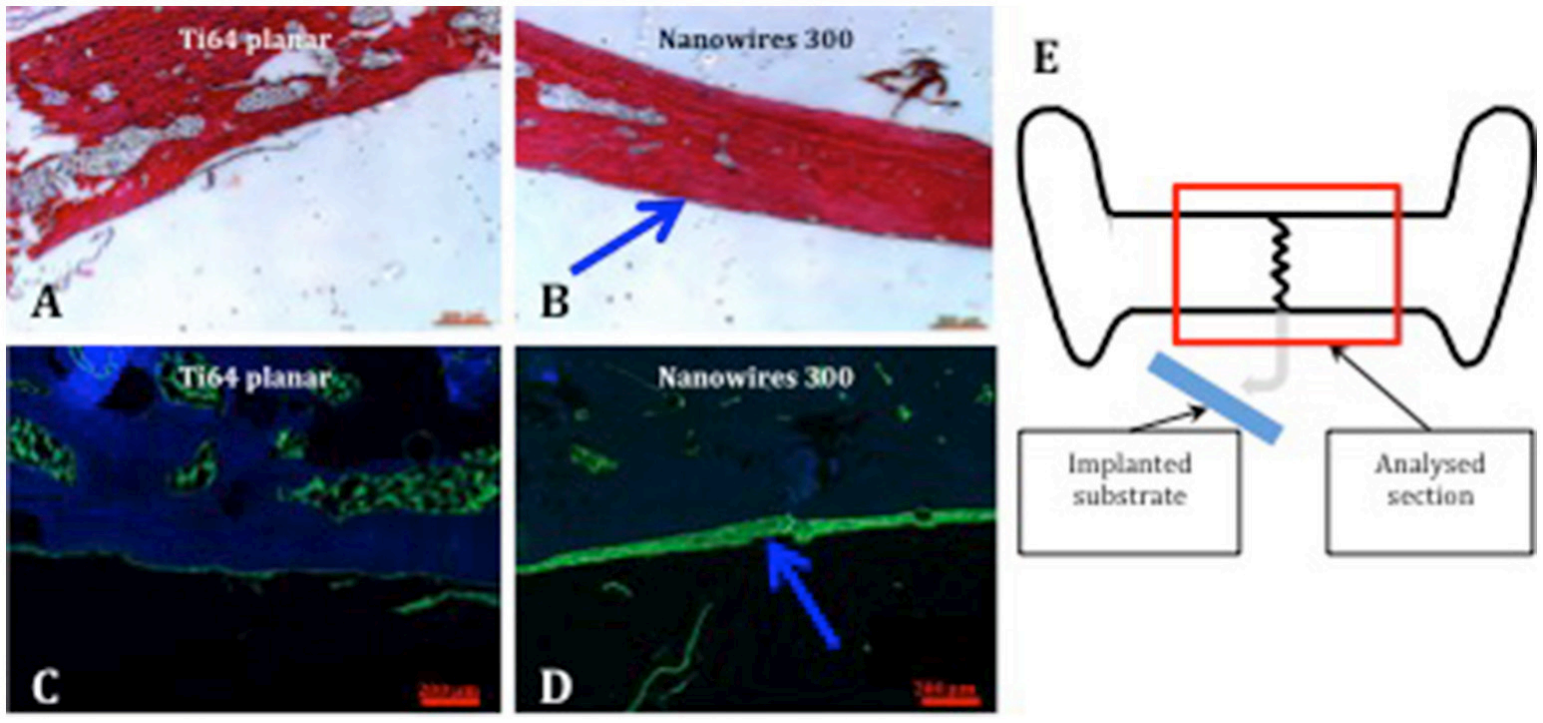
Bone formation in calvarial tissue following nanoconstruct implantation. Enhanced A&S stain and OCN immunostain of calvarial bone in contact with Ti64 planar **(A,C)** and nanowires 300 **(B,D)**, respectively. Region of de novo appositional bone formation indicated by blue arrows. OCN—green, DAPI nuclear counterstain—blue. Scale bar 200 μm. **(E)** Schematic, explaining the focus of histological tissue analysis.

Examination of proteoglycan and collagenous matrix production using Alcian blue/Sirius red (A&S) staining of calvarial bone demonstrated augmented appositional osteogenesis, evidenced as a layer of immature bone with reduced collagenous framework organization in the areas underlying the implant surfaces. This layer of *de novo* bone formation was significantly enhanced following contact with nanowire 300 topography compared to planar surfaces. Similarly, OCN immunohistochemistry revealed enhanced *de novo* appositional osteogenesis triggered by nanowire 300 topography compared to planar surfaces (Figure 7).

## Discussion

The current studies demonstrate the potential of nanowire topographies to enhance the osteogenic SSC response. Nanowire 300 topographies triggered up-regulation of bone-specific gene expression and extracellular matrix protein (OPN and Collagen type I) synthesis *in vitro*. Early topography-induced terminal lineage-specific differentiation of SSCs resulted in minimal cell proliferation and attainment of mature distinctive tear-shaped cell morphology by day 14 of *in vitro* culture on nanowires 300. This enhanced osteogenic activity *in vitro* was coupled with demonstration of significant enhancement of *de novo* contact osteogenesis at nanowire surface/bone interface within the *in vivo* rat calvarial model as revealed by CT analysis.

These findings indicate significant orthopedic potential in the application of nanowires in orthopedic implants harnessing nanowire-mediated effects in the induction of bone integration and thus implant osseointegration, and the clinical benefits therein. Interestingly, while full implant/bone contact is desirable, it has been suggested, based on 2D finite element models, that as a result of the remodeling process an equilibrium state is achieved with about 58–60% contact. This observation appears consistent with clinical observations (Lian et al., 2010). Therefore, the data from further *in vivo* and clinical trials is required to explore the potential of achieving 100% implant/bone contact with novel surface modifications.

It has previously been demonstrated that nanotopographical textures enable maintenance of SSC self-renewal and multipotency, or control differentiation (Dalby et al., 2006, 2007, 2014; McMurray et al., 2011; Kingham and Oreffo, 2013; Kingham et al., 2013; Lee et al., 2017). In our study, morphological changes occurring early in the culture process of SSCs on nanowire surfaces were correlated with differentiation of the SSCs and cessation of SSC expansion. Consistent with the low seeding density, the density of the SSCs cells remained low throughout the 21 days of *in vitro* culture, indicating minimal cell expansion as a consequence of early nanowire topography-mediated signaling and SSC differentiation. An integrated cascade of gene expression has been shown to be responsible for the modulation of the temporal osteoblast differentiation process, that includes proliferation, biosynthesis, organization and mineralization of bone extracellular matrix (Stein et al., 1996). Transition between proliferation and phenotypic differentiation occurs as a result of transcriptional shift in response to physiological regulatory signals. The physical cues of the nanowire topography resulted in modulation of SSC differentiation and activation of extracellular matrix protein synthesis. The absence of dead cells following live/dead staining, the vigorous adhesion complexes formation and cytoskeletal alignment, and strong evidence of osteogenic activity collectively indicate that nanowire surfaces were biocompatible with cell viability and function.

Interestingly, as evidenced by SEM, the formation of adhesion complexes was preceded by physical interaction of the SSC with the nanowire lattice by filopodia, enabling sensing of spatial, topographical and chemical information for the SSCs (Biggs et al., 2010; Feller et al., 2015). Thus, the physical signals involved in activation of osteoblast lineage differentiation were as a consequence of interaction of SSCs with the underlying topography by the cell filopodia with or without activation of adhesion molecules, integrins (Biggs et al., 2010; Feller et al., 2015). Cell differentiation and osteogenic induction mediated by the nanowires 50 topography, produced STRO-1 SSCs displaying the characteristic flattened osteoblast morphology. The mature elongated morphology of SSCs cultured on nanowires 300 was distinctive and unusual, and appeared to have been established by day 14 in culture with the cell projections observed to be almost twice the length of the spread cells on nanowires 50 surface. The observed morphological changes resulted from the concomitant alterations in cytoskeletal scaffolds and tensegrity. Cell phenotype and function can be regulated by transmission of physical surface signals through the adhesion-cytoskeleton-nucleus mechanotransduction pathway (Wormer et al., 2014). Actin microfilaments assembly interlinking the focal adhesion complexes is important in mediating intracellular tension and cell gene activation and bone matrix protein production (Dalby et al., 2014). Thus, cytoskeletal microfilaments form an integral link between focal adhesions and nuclear scaffolds that are essential in triggering change in the organization of cytoplasmic and nuclear molecular assemblies (Maniotis et al., 1997). In a detailed study looking at the effect of a range of topographical feature heights (150, 300, and 560 nm), widths (300, 500, and 1,000 nm) and special arrangements on human lung fibroblasts, Wang et al concluded that cellular function (i.e., Collagen type I synthesis) was dependent on the focal adhesion rearrangements modulating nuclear volume, with anisotropic arrangement of topography proving more potent due to enhanced ability to provide continuous undisrupted surface guidance (Wang K. et al., 2016). These observations are in agreement with our findings of increased cellular elongation and resultant enhanced intracellular tension found on anisotropic nanowire lattice triggering enhanced expression of osteoblast phenotype and osteogenic activity.

In the current study, a significant up-regulation in osteogenic gene induction was observed in SSCs cultured on nanowires 300 compared to Ti64 planar controls; linked to enhanced Collagen type I and Osteopontin matrix protein synthesis in comparison to control substrates. It was suggested that surfaces lacking sufficient surface roughness caused excessive cells flattening and relative impairment of nutrition (Wennerberg and Albrektsson, 2009). Thus, it is possible, the globular tear-shaped morphology of the SSCs on nanowires 300 may aid intra-cellular metabolic exchanges compared to flattened morphology on other test surfaces. The expression of bone marker genes was less robust in SSCs cultured on nanodots 10 V and nanowires 50. Interestingly, Collagen type II gene expression, characteristic of chondrogenic stimulation, was not observed further highlighting the SSCs differentiation by the nanoconstructs and the metal planar surface controls.

The characteristics of TiO_2_ nanowires vary with parameters of thermal oxidation treatment (Dinan, 2012). Previously, compared to planar controls, nanowires measuring 6 μm in length and 300 nm in diameter were shown to induce improved adhesion and proliferation, and increased ALP activity in osteosarcoma cells *in vitro* (Dinan et al., 2013). However, following demonstration of bactericidal properties of nanowires specifically measuring 3 μm in length and 20 nm in diameter, our findings represent a focused attempt at extensive characterization of nanowire osteoinductive potential within *in vitro* and *in vivo* environments using human SSCs. Harnessing *in vivo* studies, control and test samples were implanted in each individual rat to reduce the effect of physiological and anatomical variation between the rat hosts. The samples were implanted in midline with each exposed to underlying calvarial bone and the sagittal suture, thus eliminating the variation introduced by the superior regenerative potential of the stem cells derived from the suture (Maruyama et al., 2016). The same volume of bone was chosen for analysis, aided by precise alignment of the samples prior to selection of the ROI. The enhanced X-ray depletion in the nanowires 300 group was likely caused by the nanowire mesh on the sample surface contributing to the increased volume of metal material compared to controls. Consequently, x-ray depletion contributed to the higher initial (week 0) reading of bone volume in nanowires 300 group compared to the control samples. However, the effect of this shadow artifact was constant throughout the subsequent period of *in vivo* culture and therefore did not contribute further to the increase in bone volume observed in both groups. Critically, the whole thickness of an individual implant and underlying calvarial bone tissue was included in ROI to compensate for any variation in calvarial anatomy and implant placement. *In vivo* analysis demonstrated a significant increase of bone volume in contact with nanowire 300 surfaces compared to planar controls. This enhanced bone formation, a consequence of stem cell stimulation following culture on the nanowires, resulted in the augmentation of de novo appositional osteogenesis, evidenced by histological findings. The process of appositional osteogenesis occurred either through lamellar remodeling or woven bone deposition and subsequent lamellar bone substitution on the surface of the host bone bed and on the implant itself (Davies, 2003; Mavrogenis et al., 2009).

Nanowire topographies have been previously reported to display bactericidal properties (Diu et al., 2014; Sjostrom et al., 2016). Moreover, while reducing bacterial colonization, topographies fabricated using thermal oxidation were shown to support mammalian osteosarcoma cell adhesion and proliferation (Diu et al., 2014). Consistent with our findings, the proliferation of osteosarcoma cells on these surfaces was characterized by early cell isolation as opposed to monolayer formation, and distinctive morphological alterations resulting in teardrop morphology indicative of unipolar migration of cells held by topographical spikes (Diu et al., 2014). The current studies demonstrate the potential for complex surface treatment of large areas in 3D, using thermal oxidation and, critically, the potential for enhancement of implant osseointegration. The combination of bactericidal and osteoinductive properties make thermal oxidation an attractive approach for potential industrial scale application in implant fabrication.

The caveats of the current *in vitro* studies include a lack of the assessment of the scope of focal adhesion complexes-mediated cell adhesion and resultant cell deformation. The data analysis did not specifically focus on the cellular mechanisms responsible for the osteoinduction observed, which would have been to further understand and guide the material surface design. The *in vivo* study design did allow micro movement of the implanted samples, with the initial frictional interference of the nanowire surfaces offering relative protection against micro movement compared to the planar surfaces. However, both planar and nanowire surfaces achieved sufficient primary stability to enable osteogenesis as evidenced by bone volume increase in contact with both surfaces (Figure 6E). The extent of implant/bone contact and mechanical strength of the interface formed were not formally tested in these *in vivo* studies. Further research will aim to further investigate these aspects. Additionally, it was previously suggested that the concept of animal research, particularly that relating to pharmaceuticals and environmental agents, may be a poor predictor of human experience (Bracken, 2009). Therefore, although the results from *in vivo* studies demonstrated enhanced osteogenic potential of nanowires 300, a degree of caution is warranted in outcome extrapolation to the human scenario. Further steps will necessitate a comparative study including a select range of novel surface finishes identified from discrete studies as well as the current commercially available implant surface finishes. Indeed, these studies advocate thorough standardization of surface characterization parameters, cell-type and experimental design as essential. Nevertheless, the level of nanowire 300 topography-mediated enhancement in osteogenic response indicates this surface as a prominent candidate material for enhanced contact osteoinductivity.

In summary, the current studies have demonstrated an enhanced osteogenic response to nanowires 300 surface modifications, and thus the potential for augmented implant integration resulting in significant impact on clinical implant track record. This impact could be particularly relevant in areas of orthopedic implantology where clinical success and patient satisfaction levels are significantly below those observed in hip and knee replacements (i.e., shoulder, elbow, and ankle replacements). The further evaluation of nanowire 300 topography mediated enhancement of bone induction auger well for bone tissue regenerative application for an increasing aging demographic.

## Author contributions

VG, BS and RO designed the experiments. VG performed the experiments with support from GHB for in vivo and TS for materials manufacture and characterization. VG with BS, DD and RO analyzed the results. VG and RO wrote the paper, All authors approved and contributed to the final draft.

### Conflict of interest statement

The authors declare that the research was conducted in the absence of any commercial or financial relationships that could be construed as a potential conflict of interest.
